# Unappreciated

**Published:** 1873-12

**Authors:** 


### UNAPPRECIATED.

THE unappreciated living often become the glorified dead. If this could be *vice versaed* it would do some good; but as it is, what's the use? When the good man has gone to heaven what benefit is it to him that human praises are sounded to the skies, when, while here, a little of it would have lightened and lifted up his almost breaking heart, and when discouraged and baffled, and weighed down by crushing burdens, he was ready to die in very despair, without appreciation, without sympathy, the world over. Nor is this the worst side of the picture. Some of the grandest men who have ever lived, the influence of whose handiwork, to this very day, is shaping the destinies of the world for good, oftentimes pined in obscurity and neglect, were almost crushed by human heartlessness and ridicule. Columbus passed many a day in chains, and pined in noisome dungeons. Goodyear, of rubber fame, had to make a jail his home. The glorious Morse was at one time on the very point of suicide. How would a single word of cheer, and sympathy, and encouragement have lighted up their breaking hearts in these dreadful moments, when the whole sky was as black to them as midnight.

The very men who, sometimes during their earthly pilgrimage, are so bedaubed by the scurrility of their cotemporaries that they seem to be almost human monsters, have biographies made of them after death which transform them to angelic likeness. It would be a great deal better to kick the dead for their misdeeds, for, while it would not hurt them a speck, it would let the living know that wrong doing would always cloud their *post mortem* glories. On the other hand, the praises of the living, while they are struggling for the accomplishment of great ends, would be an inspiration to them, and would waft them to glorious success with redoubled speed. Let every reader, then, from this good hour, and forward to the end of his mortal life, make it a point to have a pleasant smile, a cheerful recognition, and an encouraging word for every human being whom he finds with eyes steadfast on "Excelsior."

